# Rapamycin prevents cyclophosphamide-induced ovarian follicular loss and potentially inhibits tumour proliferation in a breast cancer xenograft mouse model

**DOI:** 10.1093/humrep/deae085

**Published:** 2024-05-11

**Authors:** Yuji Tanaka, Tsukuru Amano, Akiko Nakamura, Fumi Yoshino, Akie Takebayashi, Akimasa Takahashi, Hiroyuki Yamanaka, Ayako Inatomi, Tetsuro Hanada, Yutaka Yoneoka, Shunichiro Tsuji, Takashi Murakami

**Affiliations:** Department of Obstetrics and Gynecology, Shiga University of Medical Science, Otsu, Japan; Department of Obstetrics and Gynecology, Shiga University of Medical Science, Otsu, Japan; Department of Obstetrics and Gynecology, Shiga University of Medical Science, Otsu, Japan; Department of Obstetrics and Gynecology, Shiga University of Medical Science, Otsu, Japan; Department of Obstetrics and Gynecology, Shiga University of Medical Science, Otsu, Japan; Department of Obstetrics and Gynecology, Shiga University of Medical Science, Otsu, Japan; Department of Obstetrics and Gynecology, Shiga University of Medical Science, Otsu, Japan; Department of Obstetrics and Gynecology, Shiga University of Medical Science, Otsu, Japan; Department of Obstetrics and Gynecology, Shiga University of Medical Science, Otsu, Japan; Department of Obstetrics and Gynecology, Shiga University of Medical Science, Otsu, Japan; Department of Obstetrics and Gynecology, Shiga University of Medical Science, Otsu, Japan; Department of Obstetrics and Gynecology, Shiga University of Medical Science, Otsu, Japan

**Keywords:** rapamycin, mTOR inhibitor, cyclophosphamide, breast cancer, *in vivo*, tumour bearing, fertility preservation, follicle activation, gonadotoxicity, ovarian reserve

## Abstract

**STUDY QUESTION:**

To what extent and via what mechanism does the concomitant administration of rapamycin (a follicle activation pathway inhibitor and antitumour agent) and cyclophosphamide (a highly toxic ovarian anticancer agent) prevent cyclophosphamide-induced ovarian reserve loss and inhibit tumour proliferation in a breast cancer xenograft mouse model?

**SUMMARY ANSWER:**

Daily concomitant administration of rapamycin and a cyclic regimen of cyclophosphamide, which has sufficient antitumour effects as a single agent, suppressed cyclophosphamide-induced primordial follicle loss by inhibiting primordial follicle activation in a breast cancer xenograft mouse model, suggesting the potential of an additive inhibitory effect against tumour proliferation.

**WHAT IS KNOWN ALREADY:**

Cyclophosphamide stimulates primordial follicles by activating the mammalian target of the rapamycin (mTOR) pathway, resulting in the accumulation of primary follicles, most of which undergo apoptosis. Rapamycin, an mTOR inhibitor, regulates primordial follicle activation and exhibits potential inhibitory effects against breast cancer cell proliferation.

**STUDY DESIGN, SIZE, DURATION:**

To assess ovarian follicular apoptosis, 3 weeks after administering breast cancer cells, 8-week-old mice were randomized into three treatment groups: control, cyclophosphamide, and cyclophosphamide + rapamycin (Cy + Rap) (n = 5 or 6 mice/group). Mice were treated with rapamycin or vehicle control for 1 week, followed by a single dose of cyclophosphamide or vehicle control. Subsequently, the ovaries were resected 24 h after cyclophosphamide administration (short-term treatment groups). To evaluate follicle abundance and the mTOR pathway in ovaries, as well as the antitumour effects and impact on the mTOR pathway in tumours, 8-week-old xenograft breast cancer transplanted mice were randomized into three treatment groups: vehicle control, Cy, and Cy + Rap (n = 6 or 7 mice/group). Rapamycin (5 mg/kg) or the vehicle was administered daily for 29 days. Cyclophosphamide (120 mg/kg) or the vehicle was administered thrice weekly (long-term treatment groups). The tumour diameter was measured weekly. Seven days after the last cyclophosphamide treatment, the ovaries were harvested, fixed, and sectioned (for follicle counting) or frozen (for further analysis). Similarly, the tumours were resected and fixed or frozen.

**PARTICIPANTS/MATERIALS, SETTING, METHODS:**

Terminal deoxynucleotidyl transferase dUTP nick end labelling (TUNEL) was performed to examine ovarian follicular apoptosis in the short-term treatment groups. All subsequent experiments were conducted in the long-term treatment groups. Tumour growth was evaluated using the tumour volume index. The tumour volume index indicates the relative volume, compared to the volume 3 weeks after tumour cell injection (at treatment initiation) set to 100%. Tumour cell proliferation was evaluated by Ki-67 immunostaining. Activation of the mTOR pathway in tumours was assessed using the protein extracts from tumours and analysed by western blotting. Haematoxylin and eosin staining of ovaries was used to perform differential follicle counts for primordial, primary, secondary, antral, and atretic follicles. Activation of the mTOR pathway in ovaries was assessed using protein extracts from whole ovaries and analysed by western blotting. Localization of mTOR pathway activation within ovaries was assessed by performing anti-phospho-S6 kinase (downstream of mTOR pathway) immunohistochemistry.

**MAIN RESULTS AND THE ROLE OF CHANCE:**

Ovaries of the short-term treatment groups were resected 24 h after cyclophosphamide administration and subjected to TUNEL staining of apoptotic cells. No TUNEL-positive primordial follicles were detected in the control, Cy, and Cy + Rap groups. Conversely, many granulosa cells of growing follicles were TUNEL positive in the Cy group but negative in the control and Cy + Rap groups. All subsequent experimental results were obtained from the long-term treatment groups. The tumour volume index stabilized at a mean of 160–200% in the Cy group and 130% in the Cy + Rap group throughout the treatment period. In contrast, tumours in the vehicle control group grew continuously with a mean tumour volume index of 600%, significantly greater than that of the two treatment groups. Based on the western blot analysis of tumours, the mTOR pathway was activated in the vehicle control group and downregulated in the Cy + Rap group when compared with the control and Cy groups. Ki-67 immunostaining of tumours showed significant inhibition of cell proliferation in the Cy + Rap group when compared with that in the control and Cy groups. The ovarian follicle count revealed that the Cy group had significantly fewer primordial follicles (*P* < 0.001) than the control group, whereas the Cy + Rap group had significantly higher number of primordial follicles (*P* < 0.001, 2.5 times) than the Cy group. The ratio of primary to primordial follicles was twice as high in the Cy group than in the control group; however, no significant difference was observed between the control group and the Cy + Rap group. Western blot analysis of ovaries revealed that the mTOR pathway was activated by cyclophosphamide and inhibited by rapamycin. The phospho-S6 kinase (pS6K)-positive primordial follicle rate was 2.7 times higher in the Cy group than in the control group. However, this effect was suppressed to a level similar to the control group in the Cy + Rap group.

**LARGE SCALE DATA:**

None.

**LIMITATIONS, REASONS FOR CAUTION:**

The combinatorial treatment of breast cancer tumours with rapamycin and cyclophosphamide elicited inhibitory effects on cell proliferative potential compared to cyclophosphamide monotherapy. However, no statistically significant additive effect was observed on tumour volume. Thus, the beneficial antitumour effect afforded by rapamycin administration on breast cancer could not be definitively proven. Although rapamycin has ovarian-protective effects, it does not fully counteract the ovarian toxicity of cyclophosphamide. Nevertheless, rapamycin is advantageous as an ovarian protective agent as it can be used in combination with other ovarian protective agents, such as hormonal therapy. Hence, in combination with other agents, mTOR inhibitors may be sufficiently ovario-protective against high-dose and cyclic cyclophosphamide regimens.

**WIDER IMPLICATIONS OF THE FINDINGS:**

Compared with a cyclic cyclophosphamide regimen that replicates human clinical practice under breast cancer-bearing conditions, the combination with rapamycin mitigates the ovarian follicle loss of cyclophosphamide without interfering with the anticipated antitumour effects. Hence, rapamycin may represent a new non-invasive treatment option for cyclophosphamide-induced ovarian dysfunction in breast cancer patients.

**STUDY FUNDING/COMPETING INTEREST(S):**

This work was not financially supported. The authors declare that they have no conflict of interest.

## Introduction

The primordial follicle pool is approximately assembled at birth and represents female reproductive capacity. Certain chemotherapies induce abnormal activation and depletion of primordial follicles within this fixed pool and irreversibly diminish the ovarian reserve, resulting in premature ovarian insufficiency (POI). Chemotherapy-induced infertility in the adolescent and young adult (AYA) generation is of particular concern. Breast cancer is the most common cancer among AYAs (Nakata *et al.*, 2022) with an increasing incidence (Barr *et al.*, 2016). Disease-free and overall survival outcomes in the AYA breast cancer setting are worse, with more aggressive disease and higher rates of mastectomy than in patients with adult breast cancer (Sauder *et al.*, 2020; Youlden *et al.*, 2020; Cathcart-Rake *et al.*, 2021). Since higher rates of triple-negative breast cancer and human epidermal growth factor type 2 (HER2)-positive subtypes were detected in patients with AYA breast cancer, 55.5% of patients have received chemotherapy, including neoadjuvant chemotherapy (Kataoka *et al.*, 2014). Cyclophosphamide, the most gonadotoxic anticancer drug, is frequently administered to treat breast cancer. Therefore, protecting fertility against chemotherapy-induced ovarian dysfunction is critical, and the prevention of cyclophosphamide-induced ovarian dysfunction in patients with breast cancer warrants further study.

There are three fertility preservation strategies in female patients with cancer: frozen embryo/egg preservation, ovarian tissue cryopreservation and transplantation (Diaz-Garcia *et al.*, 2018; Dolmans *et al.*, 2021), and ovarian protection with drugs (Roness *et al.*, 2016). Drug-assisted ovarian protection is a valuable option as it can be used in combination with either of the other two methods and has no noticeable disadvantages; however, no drug with a high level of supportive evidence has been reported. Currently, the GnRH agonists have accumulated the most evidence as ovarian protective agents. However, the European Society for Medical Oncology (ESMO) (Lambertini *et al.*, 2020) and American Society of Clinical Oncology (ASCO) (Oktay *et al.*, 2018) guidelines emphasize that GnRH agonists are a therapeutic option but not a substitute for embryo/oocyte freezing and are recommended for use in situations where these are not available. The ESHRE Guideline Group on Female Fertility Preservation (2020) similarly states that GnRH agonists, although a treatment option, have limited protective effects on ovarian reserve and future fertility. Although several randomized controlled trials have shown the efficacy of GnRH agonists (Moore *et al.*, 2015), others have failed to demonstrate efficacy (Munster *et al.*, 2012), whereas others have not shown efficacy but argue limited effectiveness (Leonard *et al.*, 2017). Hence, ovarian protective agents other than GnRH analogues are eagerly anticipated in clinical practice.

A breakthrough discovery was made regarding chemotherapy-induced ovarian dysfunction: cyclophosphamide induces ovarian damage in part by activation of the mammalian target of the rapamycin (mTOR) pathway, leading to primordial follicle activation and follicular ‘burnout’ (Adhikari and Liu, 2010). Based on this accumulated evidence, we previously reported the ovarian protective effects of mTOR inhibitors (Tanaka *et al.*, 2018). Others have supported our supposition on the ovarian protective effect of mTOR inhibitors (Goldman *et al.*, 2017; Zhou *et al.*, 2017; Xie *et al.*, 2020; Chen *et al.*, 2022a; Kashi *et al.*, 2023). Meanwhile, mTOR inhibitors have several other effects (Li *et al.*, 2014); in terms of safety, mTOR inhibitors have proven relatively safe long-term oral agents in human solid organ transplantation and tuberous sclerosis (Somers and Paul, 2015; Ventura-Aguiar *et al.*, 2016). Notably, mTOR inhibitors also reportedly elicit antitumour effects in breast cancer (Zeng *et al.*, 2010; Baselga *et al.*, 2012).

The greatest challenge facing existing research on protective agents against chemotherapy-induced ovarian hypofunction is the relative lack of reports in cancer-bearing mice. The most common approach for studying ovarian-protective agents is to administer chemotherapeutic and ovarian-protective agents to non-cancerous mice. However, chemotherapy dosage regimens have not been studied based on antitumour efficacy but on the basis of chemotherapy-induced ovarian dysfunction; that is, unlike real-world clinical practice, chemotherapy was administered as a single dose or cyclical administration of low doses. Furthermore, since the subject mice are not carcinoma bearing, it is unclear whether cyclophosphamide administration sufficiently reproduces antitumour effects. Moreover, the lack of observed effects elicited by ovarian protective agents on tumours has proven problematic.

Accordingly, the primary objective of the present study was to develop a breast cancer mouse model that is cyclically administered cyclophosphamide (a potent standalone antitumour agent) and simultaneously provided an ovarian protective agent, rapamycin (an mTOR inhibitor known for its antitumour properties). Subsequent analyses assessed the combined effects of these treatments on ovarian protection and breast cancer antitumour activity.

## Materials and methods

### *In vivo* xenograft breast cancer model

All procedures were approved by the Institutional Animal Care and Use Committee (approval number: 2022-6-16). Female BALB/c nude mice at the age of 4 weeks were purchased from SLC (Japan), housed under standard conditions with a 12-h light/12-h dark cycle, and fed standard pelleted rodent chow. The sample size was determined based on a previous *in vivo* study with rapamycin and cyclophosphamide co-treatment in breast cancer (Zeng *et al.*, 2010). The mice were acclimatized for 1 week. MDA-MB-231 human mammary adenocarcinoma cells, expressing low-level HER2, also express low levels of oestrogen and progesterone receptors and are highly invasive. MDA-MB-231 cells were purchased from the American Type Culture Collection (ATCC, USA). Cells were cultured in Dulbecco’s modified eagle medium (DMEM) supplemented with 10% foetal calf serum (FCS), l-glutamine, and penicillin/streptomycin solution (all Gibco Invitrogen, USA) in a humidified 5% CO_2_, 95% ambient air atmosphere at 37°C*.* An *in vivo* xenograft breast cancer model was established by injecting 5 × 10^6^ MDA-MB-231 cells (in 100 μl of DMEM) into the mouse flank subcutaneously.

### Short-term treatment protocol for evaluating ovarian follicle apoptosis

**Figure 1.**
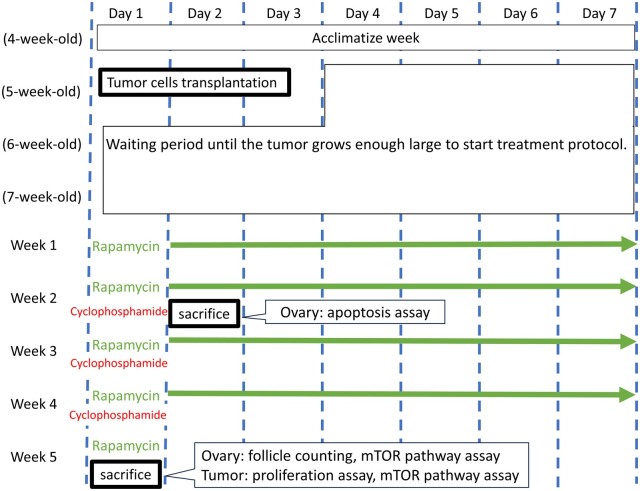
**Experimental schema: tumour cells were transplanted into 5-week-old mice, and 3 weeks after transplantation (at 8 weeks of age), the treatment protocol was initiated**. Rapamycin (green) at a dosage of 5 mg/kg, or its vehicle, was administered daily from treatment Week 1, Day 1, through Week 5, Day 1. Cyclophosphamide (red) at a dosage of 120 mg/kg, or its vehicle, was administered weekly from treatment Week 2, Day 1, through Week 4, Day 1. To analyse ovarian follicle apoptosis, mice were sacrificed on treatment Week 2, Day 2, i.e. 24 h after a single cyclophosphamide administration. To perform follicle counting and assess the mTOR pathway in the ovary, as well as the antitumour effects, mice were sacrificed on treatment Week 5, Day 1, i.e. 7 days after the last cyclophosphamide administration.

In brief, 5 × 10^6^ MDA-MB-231 cells were injected into the mouse flank subcutaneously. To replicate AYA generation in human clinical practice, the treatment protocol was initiated when the mice were 8 weeks old (3 weeks after tumour cell injection). Eight-week-old mice are considered to approximate 20- to 30-year-old humans (Dou *et al.*, 2017). Three weeks after tumour injection, mice were randomized into three treatment groups: vehicle group (control), cyclophosphamide-only group (Cy), and cyclophosphamide + rapamycin combination group (Cy + Rap) (n = 5–6/group). Cyclophosphamide (FUJIFILM, Japan) was dissolved in phosphate-buffered saline (PBS); rapamycin (FUJIFILM, Japan) supplied in dimethyl sulfoxide (DMSO) at −20°C was diluted in PBS before use. Additionally, 5 mg/kg of rapamycin or vehicle (DMSO in PBS) was intraperitoneally administered daily until sacrifice. After 7 days of rapamycin administration, a single 120 mg/kg dose of cyclophosphamide or vehicle control was administered intraperitoneally. Mice were sacrificed 24 h after cyclophosphamide administration. The ovaries were harvested and immediately submerged in 4% (v/v) paraformaldehyde (PFA) and analysed using the Terminal deoxynucleotidyl transferase dUTP nick end labelling (TUNEL) assay (Fig. 1).

### Long-term treatment protocols for evaluating follicle and tumour development

In brief, 5 × 10^6^ MDA-MB-231 cells were injected into the mouse flank subcutaneously (n = 20 mice). Three weeks after tumour injection, 19 mice with palpable tumours were randomly divided into three groups (n = 6 or 7/group): control, Cy, and Cy + Rap groups. Rapamycin (5 mg/kg) or vehicle was administered intraperitoneally (daily) for 29 days. Additionally, 120 mg/kg cyclophosphamide or vehicle was administered intraperitoneally (weekly) on Day 1 of Weeks 2–4. Mice were euthanized on Day 1 of Week 5. Considering that steady-state mTOR inhibitor concentrations are not reached for ∼7 days (Kirchner *et al.*, 2004), we administered mTOR inhibitors for 7 days before the first exposure to chemotherapy.

The treatment protocol was designed based on previously reported data and protocols. In mouse xenograft models of human breast cancer, the standard cyclophosphamide treatment regimen typically involves a weekly 100 mg/kg dosage (Tongu *et al.*, 2010; Proia *et al.*, 2014; Himmel *et al.*, 2016; Guido *et al.*, 2021). Meanwhile, a single cyclophosphamide dose of 75–150 mg/kg or repeated 75 mg/kg/week dose reportedly induce subfertility in rodent studies (Kalich-Philosoph *et al.*, 2013; Li *et al.*, 2014; Goldman *et al.*, 2017; Zhou *et al.*, 2017; Bellusci *et al.*, 2019; Luan *et al.*, 2019; Yang *et al.*, 2021; Chen *et al.*, 2022a,b; Kashi *et al.*, 2023). Thus, to demonstrate the potential of rapamycin to preserve ovarian function during cyclophosphamide monotherapy regimens, which exert sufficient antitumour effects as a single agent, we selected higher cyclophosphamide doses (i.e. total dose: 360 mg/kg, weekly dose: 120 mg/kg). In contrast, our rapamycin dosage followed standard anti-cancer (Zeng *et al.*, 2010; Weng *et al.*, 2014; Zhou *et al.*, 2016) and ovarian protection (Zhou *et al.*, 2017; Xie *et al.*, 2020; Chen *et al.*, 2022a) protocols. Tumour volume was monitored weekly by measuring two perpendicular tumour diameters with a calliper and was calculated using the following formula:

$$\text{Tumour}\;\text{volume}\;\left({\text{mm}}^{3}\right)=\left(\text{length}\;\left[\text{mm}\right]\right)\times {\left(\text{width}\;\left[\text{mm}\right]\right)}^{2}\;\times \;0.52.$$

In cancer xenograft models, treatment protocols are typically initiated when the tumour volume reaches a predetermined value after tumour transplantation. Accordingly, the age of mice at treatment initiations can vary considerably among studies. However, in studies on ovarian dysfunction, age-related ovarian dysfunction must be excluded; therefore, the number of weeks following tumour cell transplantation and before initiating treatment was fixed in the present study. Tumour growth was evaluated using tumour volume index rather than absolute tumour volume after confirming that no significant differences existed in the absolute tumour volume between the groups at treatment initiation. Tumour volume index indicates the relative volume, compared to the volume 3 weeks after tumour cell injection (at treatment initiation) set to 100%. The body weights of the mice were recorded daily.

At the time of sacrifice, the ovaries and tumours were harvested and immediately immersed in 4% PFA or liquid nitrogen (Fig. 1). The follicle count assay was performed on ovaries immersed in PFA via haematoxylin and eosin (HE) staining; the mTOR pathway assay was performed based on phospho-S6 kinase (pS6K) immunohistochemistry. Meanwhile, the proliferation of cells in tumours immersed in PFA was analysed based on Ki-67 immunohistochemistry. The ovaries and tumours immersed in liquid nitrogen were analysed for mTOR activation by western blotting.

### Ovarian follicular apoptosis assay (TUNEL immunostaining)

The commercial TUNEL assay In Situ Cell Death Detection Kit-POD (Roche, Switzerland) was employed to evaluate follicle cell apoptosis. After dewaxing and rehydrating, the sections were pretreated with proteinase K and immersed in 3% H_2_O_2_. Next, 50 µl of TUNEL working solution was added to each sample and mixed with label solution and enzyme solution; negative controls were treated with label solution. The sections were incubated at 37°C for 60 min in the dark and analysed by diaminobenzidine (DAB) staining. If more than 50% positive cells and/or positive oocytes were present, the primordial follicle was defined as being in an apoptotic state. As a positive control, a paraffin-embedded ovarian section treated with DNase I (Takara Bio, Japan)was appropriately stained.

### Ovarian follicle classification and count

**Figure 2.**
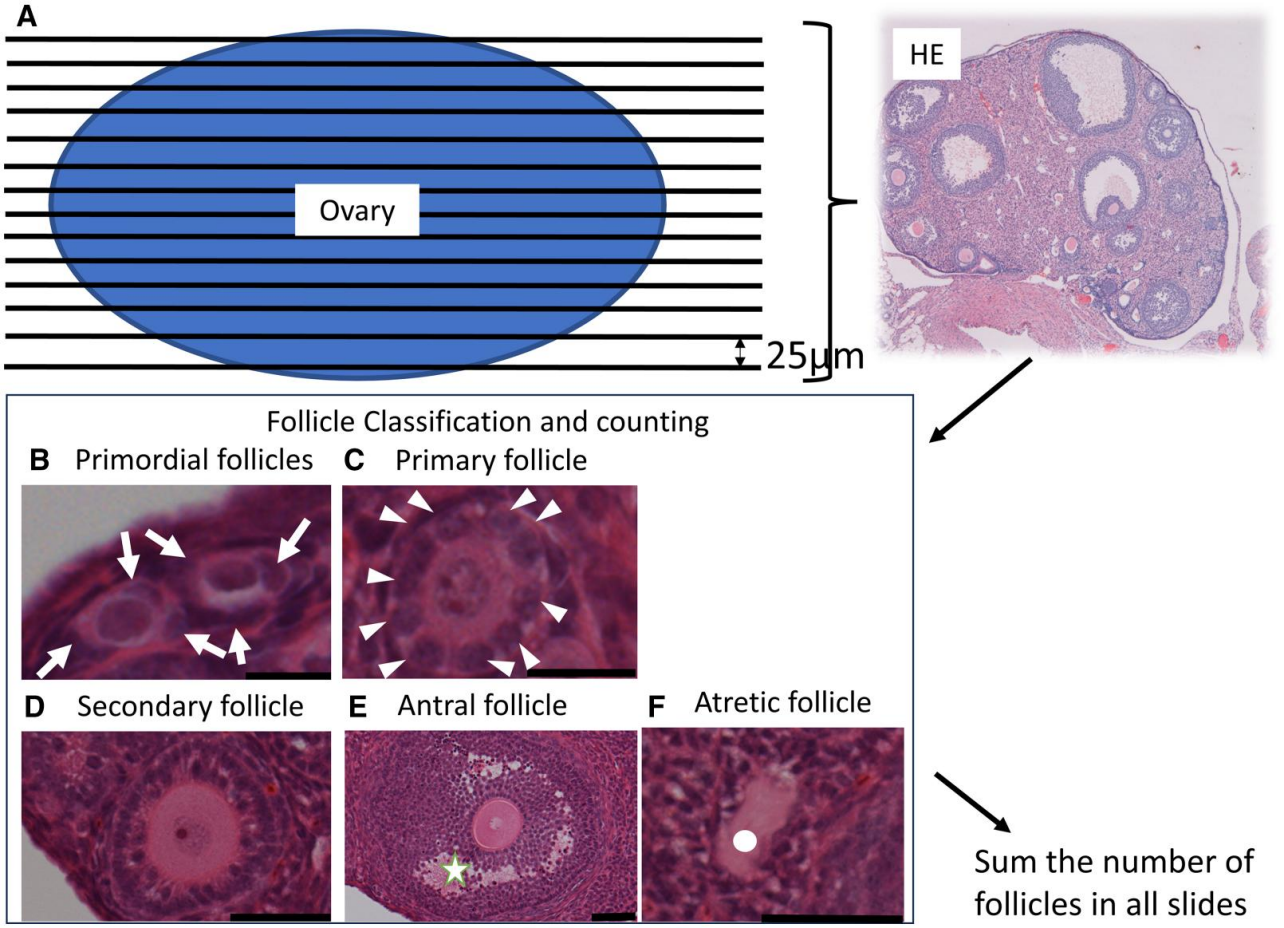
**Follicle count method**. (**A**) The ovaries were placed horizontally in the long-axis direction and paraffin-embedded so that the cross-sectional area would be as large as possible when cut. Ovaries were cut every 25 µm from end to end into 5- µm thickness sections and were rehydrated and stained with haematoxylin and eosin (HE) for morphological observation and differential follicle counts. Follicle classification and counting were performed blindly on all sections of the ovaries, and the results were then summed up across all sections. (**B**) Primordial follicle oocytes were surrounded by a single layer of squamous granulosa cells (arrows). Bar = 10 μm. (C) A primary follicle was defined as an oocyte surrounded by a single layer of cuboidal granulosa cells (arrow heads). Bar = 20 μm. (**D**) A secondary follicle was defined by two or more layers of cuboidal granulosa cells with no antrum. Bar = 50 μm. (**E**) Antral follicle was defined by two or more layers of cuboidal granulosa cells with antral cavity (star). Bar = 100 μm. (**F**) Zona pellucida remnants representing end-stage atretic follicles (circle); Bar = 100 μm.

The ovaries were fixed in 4% PFA for 24 h, dehydrated, placed horizontally along the long-axis, and paraffin-embedded such that the cross-sectional area was as large as possible when cut. The whole ovaries were sliced every 25 µm into 5-µm sections and follicles were enumerated for the entire ovary. The 25-μm serial sections were rehydrated and HE-stained for morphological observation and differential follicle counts. Blind follicle classification and counting were performed on all ovarian sections; the totals for all sections were summed. Differential follicle counts were performed, and the follicle stage was classified according to accepted published definitions (Fig. 2) (Myers *et al.*, 2004). A follicle was defined as primordial if it was surrounded by a single layer of squamous granulosa cells. A primary follicle was an oocyte surrounded by a single layer of cuboidal granulosa cells. Secondary follicles were defined as two or more layers of cuboidal granulosa cells without an antrum. Antral follicles are defined as two or more layers of cuboidal granulosa cells within the antral cavity. Only follicles containing an oocyte were counted to prevent double follicle counts. Zona pellucida remnants represented atretic follicles. The ratio of primary to primordial follicles was calculated by dividing the total number of primary follicles in each treatment group by the total number of primordial follicles in each treatment group. The minor axis index of the ovary was defined as the number of sections in which ovarian tissue was observed in consecutive sections.

### Ovarian follicular mTOR pathway activation assay (pS6K immunostaining)

pS6K is a downstream serine/threonine kinase of mTOR (Goldman *et al.*, 2017). Localization of mTOR pathway activation within the ovary was assessed by pS6K immunohistochemistry. Using the fragment next to the largest diameter observed in the HE-stained ovarian specimen, sections were cut and stored as representative sections for immunostaining. Paraffin-embedded ovarian sections were warmed and serially de-paraffinized in xylene and ethanol, incubated in EDTA buffer (pH 9.0), heated in a microwave for 10 min, and immersed in 3% H_2_O_2_ for 10 min at room temperature to block endogenous peroxidase activity. Nonspecific binding was blocked with an antigen-unmasking solution for 20 min at room temperature. The sections were incubated in a 1:50 dilution of the primary antibody phospho-rpS6 (9205; Cell Signalling Technology, USA) overnight at 4°C. For immunohistochemistry, sections were incubated with appropriate secondary antibodies for 2 h at room temperature, followed by DAB staining. The proportion of pS6K-positive cells among the primordial follicles was quantified (n = 4–5/group). As a positive control, a paraffin-embedded kidney section was appropriately stained.

### Tumour cell proliferation assay (Ki-67 immunostaining)

Tumour cell proliferation was visualized via anti-Ki-67 immunohistochemistry (1:400) as previously described in an *in vivo* breast cancer study (Namba *et al.*, 2006; Zeng *et al.*, 2010; Macaskill *et al.*, 2011). Three representative fields of view for tumours excised from each group (n = 2–3/group) were analysed under 40× magnification. Ki-67-positive nuclei were quantitated as percentages of the total nuclei. The average of three fields of view served as the cell proliferation index for the tumours. As a positive control, a paraffin-embedded spleen section was appropriately stained.

### Ovary and tumour mTOR pathway activation assay (western blotting)

Mouse ovaries were pulverized, subsequently extracted by radio-immunoprecipitation assay buffer (Nacalai Tesque Inc., Japan), and centrifuged at 10 000 × *g* at 4°C for 10 min. The supernatant was collected and stored at −80°C until western blotting was performed. The protein content was determined using a bicinchoninic acid assay (BCA) protein assay kit (Thermo Fisher Scientific Inc., USA); 15 μg of the protein from each sample was loaded onto 4–20% polyacrylamide gel for sodium dodecyl sulphate polyacrylamide gel electrophoresis. Proteins of interest were separated by electrophoresis and transferred to polyvinylidene fluoride membranes. The membranes were blocked in 3% bovine serum albumin solution for 1 h and probed with specific primary antibodies overnight at 4°C. Primary antibodies against mTOR (# 2983), phospho-mTOR (Ser2448 # 2971), S6K (# 2708), phospho-S6K (Thr389 # 9205), 4E-BP1 (rabbit mAb 9644), phospho-4E-BP1 (rabbit mAb 9451), and β-actin (# 4970) were all rabbit monoclonal antibodies purchased from Cell Signalling Technology (USA). Horseradish peroxidase-conjugated goat anti-rabbit IgG (7074, CST) was used to detect proteins, and a Chemi-Lumi One Super kit (Nacalai Tesque Inc., Japan) was employed to visualize the bands. β-Actin expression was measured as a loading control.

### Statistical analysis

The results were analysed using the JMP version 17 software (JMP, USA). A one-way ANOVA was used where appropriate. Data are presented as mean ± SEM. Differences among groups were tested using ANOVA. If an overall significant difference was found, a post hoc Tukey’s test was performed for multiple comparisons. Statistical significance was set at *P* < 0.05.

## Results

### Rapamycin and cyclophosphamide treatment do not induce obvious adverse effects

**Figure 3.**
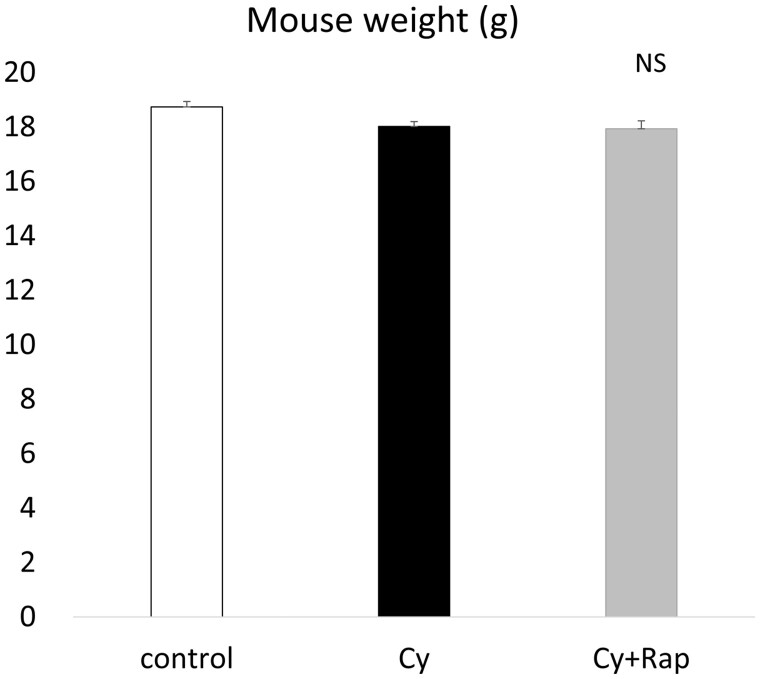
**Final body weights of the mice**. There were no statistically significant differences in body weight in any group, despite repeated cyclophosphamide (Cy) and rapamycin (Rap) administration over a long period. Data represent the mean ± SEM values.

Treatment with 5 mg/kg/day rapamycin for 29 days with or without three cycles of 120 mg/kg/week cyclophosphamide did not elicit obvious abnormalities at necropsy. No significant weight losses were observed among the mouse groups (Fig. 3).

### Antitumour effects of cyclophosphamide and rapamycin in mouse xenograft breast cancer model

**Figure 4.**
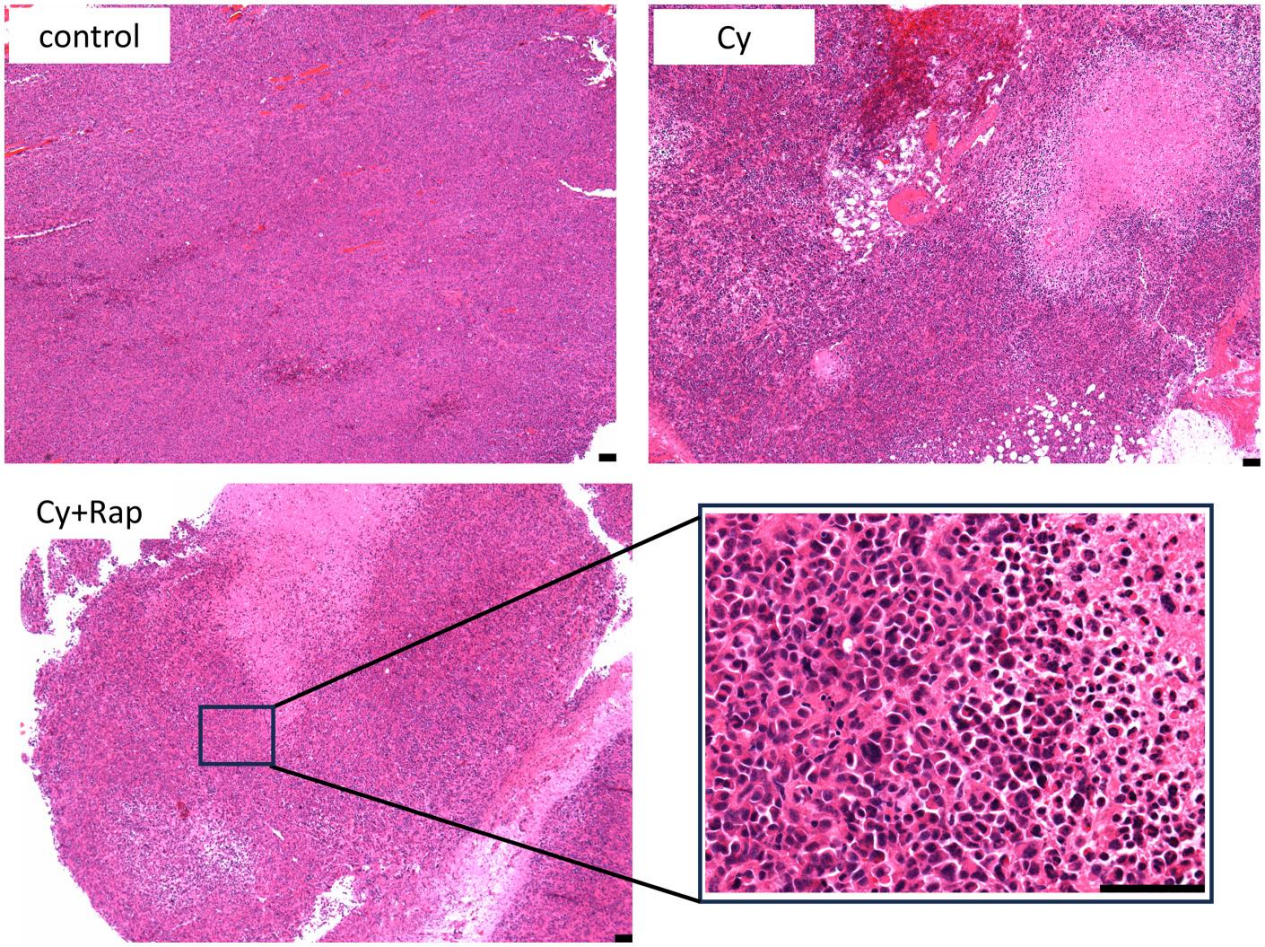
**Haematoxylin and eosin (HE) staining of tumours**. Typical HE staining of the tumours in each group is shown. Bar = 100 μm. Cy, cyclophosphamide; Rap, rapamycin.

**Figure 5.**
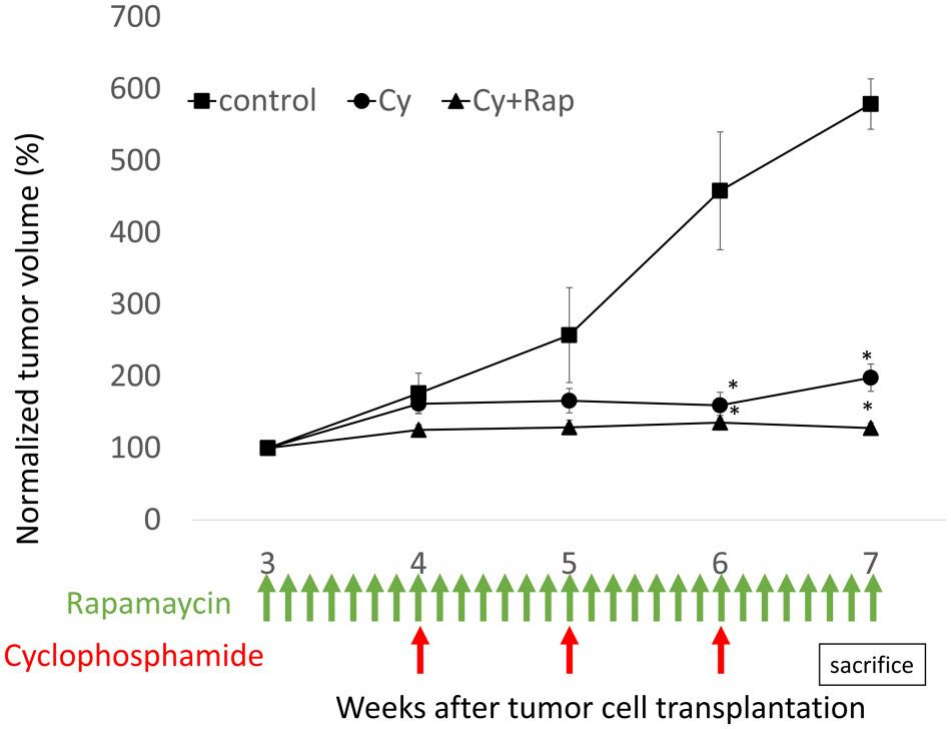
**Antitumour effects of cyclophosphamide and rapamycin in mouse xenograft breast cancer model**. Subcutaneous transplantation tumour model in nude BALB/c mice was established using the human breast cancer cell line MDA-MB-231. The mice were divided into control, Cy, and Cy + Rap groups with 6 or 7 mice per group. Treatment protocol schema and the growth curve of normalized tumour volume are shown: *P < 0.001 compared with the control. All data represent the mean ± SEM values. Cy, cyclophosphamide; Rap, rapamycin.

HE-stained tumours showed no necrosis in the control group, whereas extensive necrosis was observed in the Cy and Cy + Rap groups (Fig. 4). During the first week of treatment, the vehicle control and Cy groups, both administered the placebo, exhibited similar tumour volume indices (mean: 176% and 161%, respectively). In contrast, the Cy + Rap group, administered daily rapamycin, had a tumour volume index of 125%. After the second week of treatment (4 weeks after cell transplantation), the tumour volume index for the Cy group (weekly cyclophosphamide monotherapy) and Cy + Rap group (cyclophosphamide weekly + rapamycin daily) stabilized at a mean of 160–197% and 130%, respectively, until the end of treatment. In contrast, tumours in the control group grew steadily, with a mean tumour volume index of 578% 4 weeks after treatment initiation. Statistically significant differences in the final tumour volume index were observed between the vehicle control and both treatment groups (Fig. 5).

**Figure 6.**
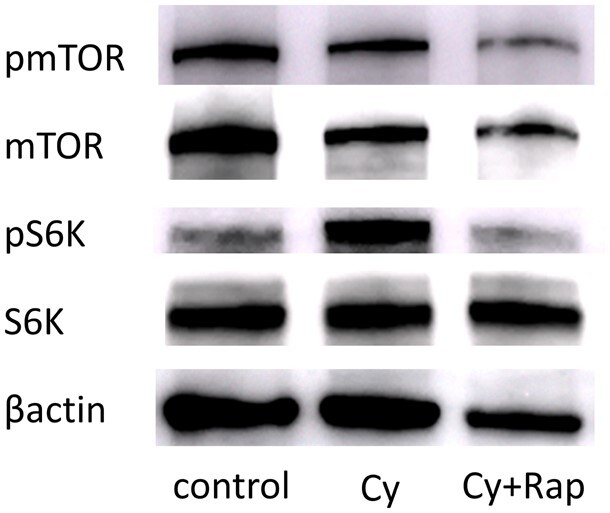
**Rapamycin inhibits mTOR pathway in mouse xenograft breast cancer model**. Representative western blots from tumour xenograft tissue are shown. Phosphorylation of mTOR and S6K was inhibited by rapamycin treatment (Cy + Rap) than in the control or Cy groups. Cy, cyclophosphamide;; pmTOR, phospho mTOR; pS6K, phospho S6K; Rap, rapamycin; S6K, S6 kinase.

**Figure 7.**
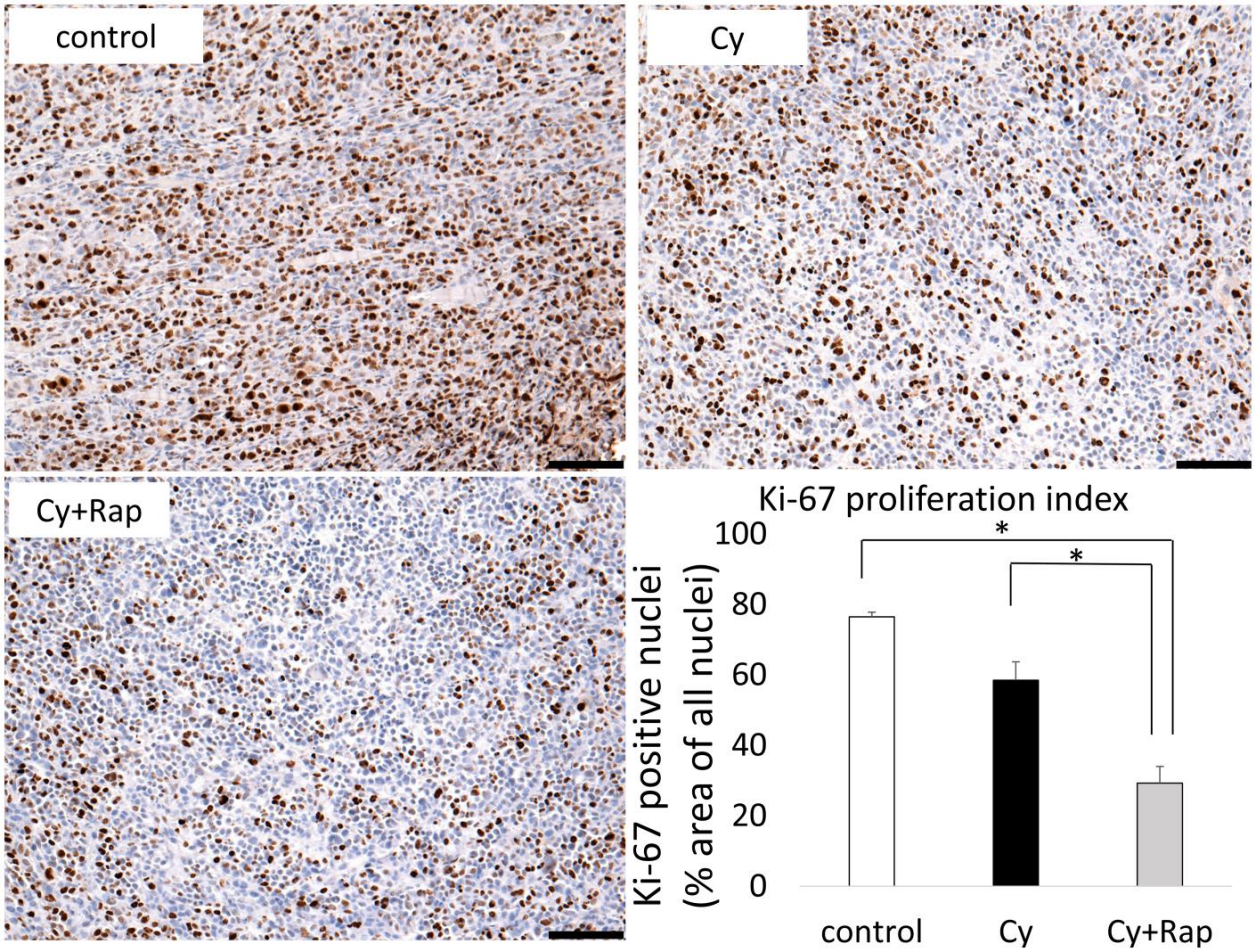
**Inhibitory effect of rapamycin and cyclophosphamide on tumour cell proliferative activity**. The tumour proliferation inhibitory effect of rapamycin was evaluated using Ki-67 immunostaining. Brown indicates Ki-67 stain-positive cells, while blue indicates Ki-67 stain-negative cells. The Cy + Rap group had a significantly lower Ki-67 positive cell rate than the Cy and control groups. *P* < 0.01, bar = 100 μm. The data represent the mean ± SEM values. Cy, cyclophosphamide; Rap, rapamycin.

Western blotting revealed that phospho mTOR (pmTOR) and pS6K were expressed in the control group; high mTOR expression in human clinical triple-negative breast cancer was reproduced in our *in vivo* xenograft model. Phosphorylation of mTOR and S6K was inhibited more significantly by rapamycin treatment than in the control or Cy groups (Fig. 6). Moreover, the Cy + Rap group exhibited markedly fewer Ki-67-positively stained cells than the Cy and control groups, indicating decreased tumour cell proliferation (*P* < 0.01; Fig. 7).

### Short-term treatment with cyclophosphamide does not induce apoptosis in primordial follicles but can induce apoptosis in growing follicles and this effect is inhibited by rapamycin

**Figure 8.**
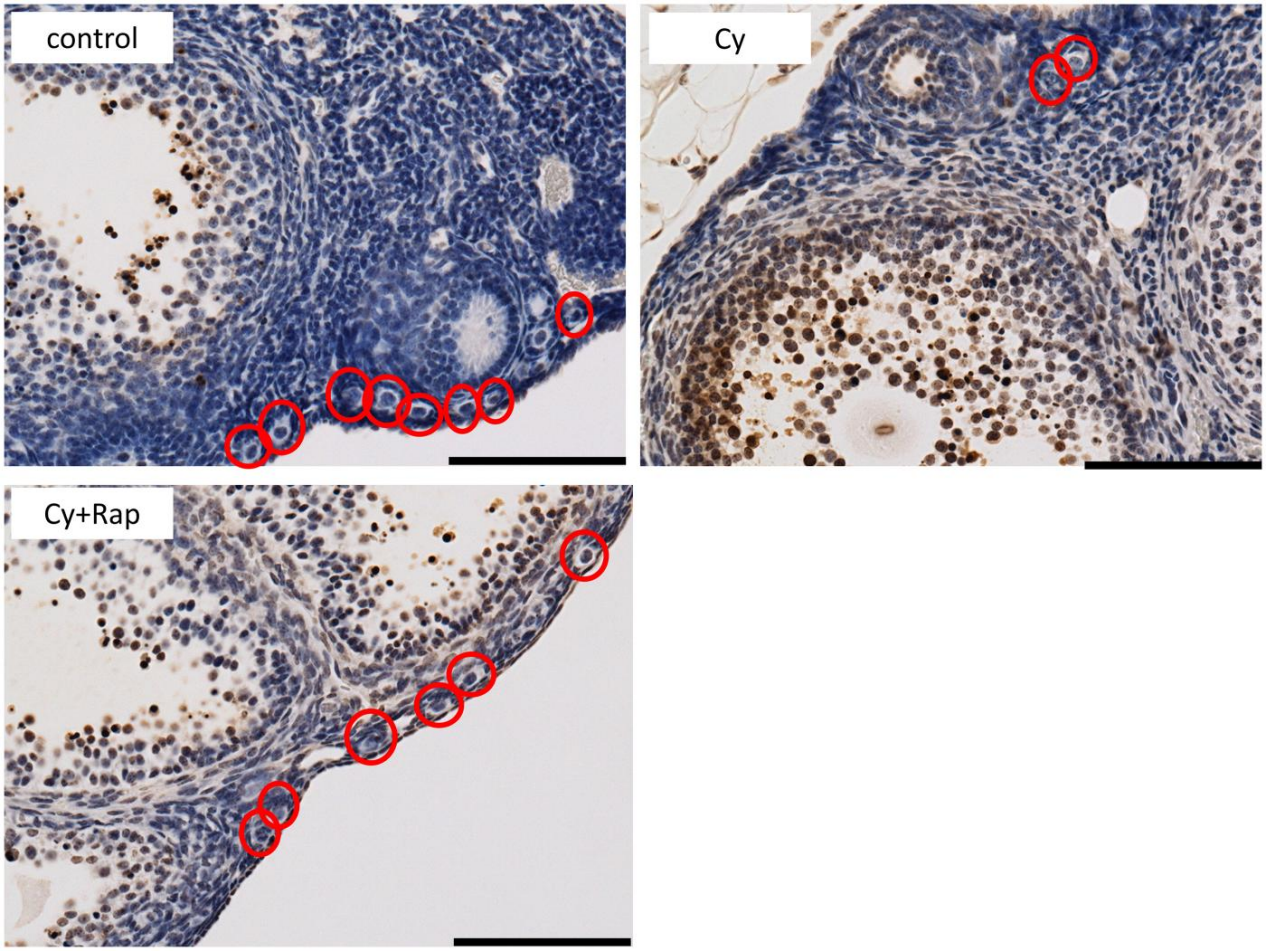
**Terminal transferase dUTP nick end labelling (TUNEL) staining of ovaries resected 24 h after cyclophosphamide administration**. Brown indicates TUNEL stain-positive cells, while blue indicates TUNEL stain-negative cells. No TUNEL stain-positive primordial follicles can be observed in the control, Cy, and Cy + Rap groups. All primordial follicles in the control, Cy, and Cy + Rap groups were negative for TUNEL staining (indicated by red circles). Conversely, numerous granulosa cells of growing follicles were TUNEL positive in the Cy group and relatively negative in the control and Cy + Rap groups. Scale bar = 100 μm. Cy, cyclophosphamide; Rap, rapamycin.

Based on the TUNEL staining of ovaries resected 24 h after short-term cyclophosphamide administration, no positively stained primordial follicles were detected in the control, Cy, or Cy + Rap groups. Conversely, numerous granulosa cells within growing follicles were TUNEL positive in the Cy group but relatively negative in the control and CY + Rap groups (Fig. 8).

### Protective effect of rapamycin as an mTOR inhibitor on the *in vivo* model of long-term cyclophosphamide-induced ovarian follicle loss

**Figure 9.**
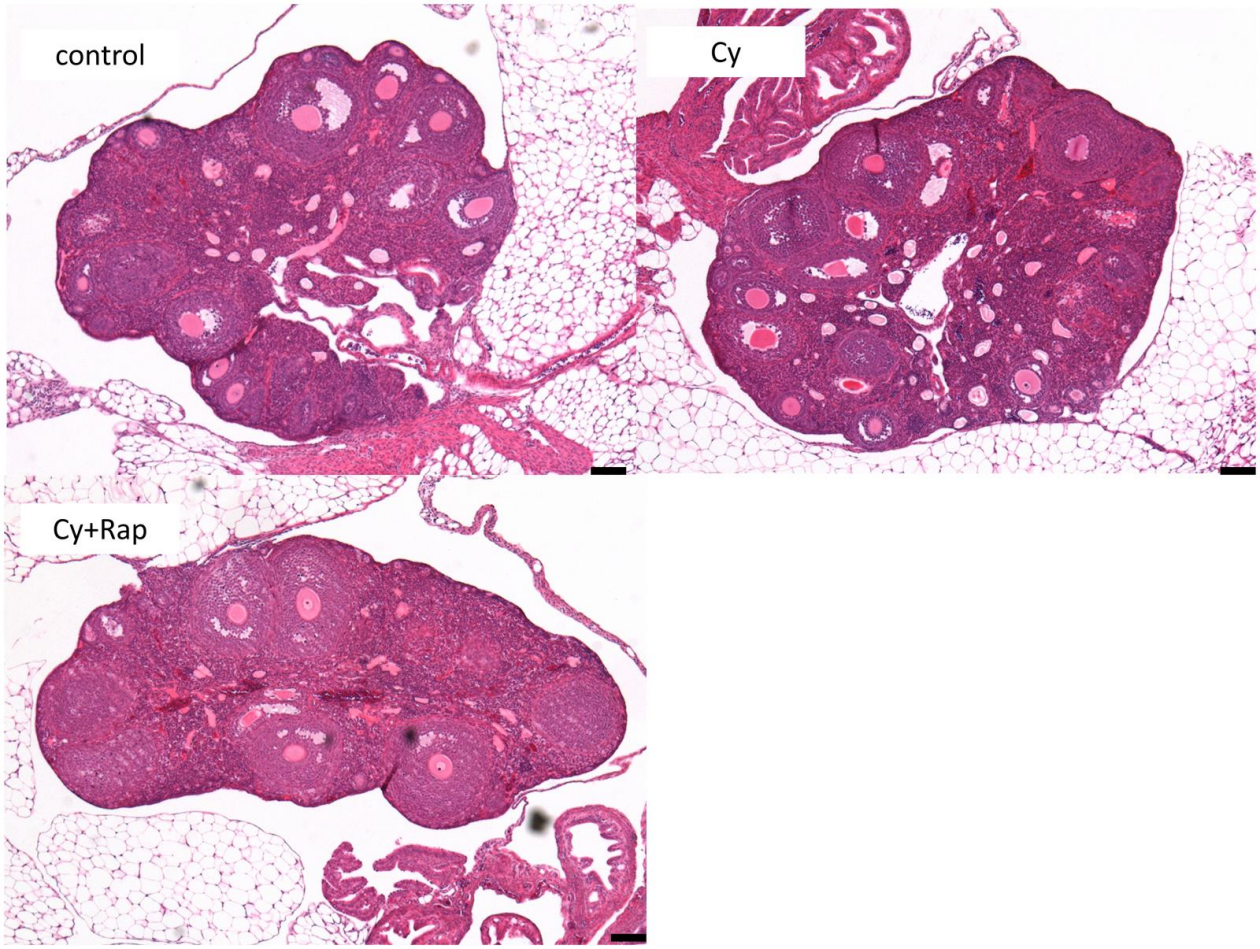
**Haematoxylin and eosin (HE) staining histological examination of ovaries**. Low-magnification HE staining revealed that the Cy and Cy + Rap groups exhibited no destruction of the ovarian structure, such as stromal fibrosis despite cyclic cyclophosphamide administration. the Cy + Rap group showed a reduced growing follicle count (e.g. secondary and antral follicles); scale bar = 100 μm. Cy, cyclophosphamide; Rap, rapamycin.

Low-magnification histological analysis found no obvious morphological differences in the stroma of the control group and the Cy and Cy + Rap groups (Fig. 9). We also investigated the effects of the mTOR inhibitor (rapamycin) and cyclophosphamide on the number of follicles in each group. The ovarian tissue of the Cy group had significantly fewer primordial follicles (*P* < 0.001) than the control group. Meanwhile, the ovarian tissue of the Cy + Rap group had significantly more (∼2.5 times) primordial follicles (*P* < 0.001) than the Cy group. That is, the Cy + Rap group had 62% of the primordial follicles observed in the control group, whereas the Cy group had 26%. This indicates that the addition of rapamycin can inhibit cyclophosphamide-induced primordial follicle loss.

There was also a statistically significant difference in the number of primordial follicles between the control and Cy + Rap groups (*P* < 0.001). The Cy + Rap group had fewer growing (secondary and antral) follicles than the control groups. Meanwhile, the atretic follicle count did not differ significantly among all groups. The Cy + Rap group also had significantly smaller ovaries than the control group (*P* < 0.05), indicating that there were fewer secondary and antral follicles, which contribute extensively to the ovary size, in the Cy + Rap group compared with the control group.

**Figure 10.**
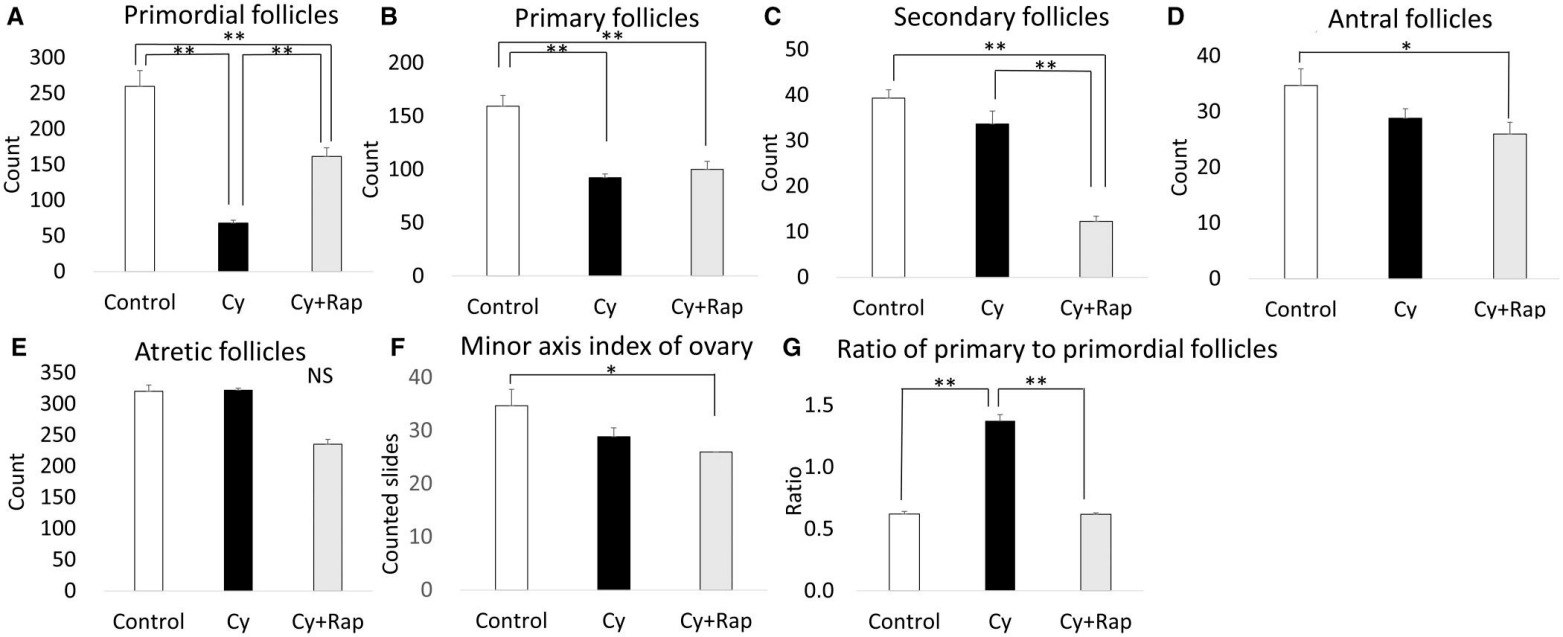
**Follicle counts**. (**A**) Primordial follicle count: the Cy group had significantly fewer primordial follicles than the control group. The Cy + Rap group had significantly more primordial follicles than the Cy group. Cy, cyclophosphamide; Rap, rapamycin. (**B**) Primary follicle count: the Cy and Cy + Rap groups had significantly fewer primary follicles than the control group. (**C**) Secondary follicle count: the Cy + Rap group had significantly fewer secondary follicles than the control and Cy groups. (D) Antral follicle count: the Cy + Rap group had significantly fewer antral follicles than the control group. (**E**) Atretic follicle count: No statistically significant differences were observed. (**F**) Minor axis index of ovary: the minor axis index of the ovary was defined as the number of sections in which ovarian tissue was observed in consecutive sections. The Cy + Rap group had significantly lower minor axis index of ovary than the control group. (**G**) Ratio of primary to primordial follicles: ovaries of Cy-treated mice had over twice the ratio of primary to primordial follicles compared with all other treatment groups. Ovaries of mice cotreated with Cy + Rap had ratios of primary to primordial follicles matching untreated controls. *P < 0.05, **P < 0.001. All data represent the mean ± SEM values.

The ratio of primary to primordial follicles in the Cy group was twice that of the control group. Meanwhile, the addition of rapamycin (the Cy + Rap group) reduced this ratio to a level that did not differ significantly from the control group (Fig. 10). This indicates that rapamycin can prevent cyclophosphamide from stimulating excessive differentiation of primordial follicles into primary follicles.

**Figure 11.**
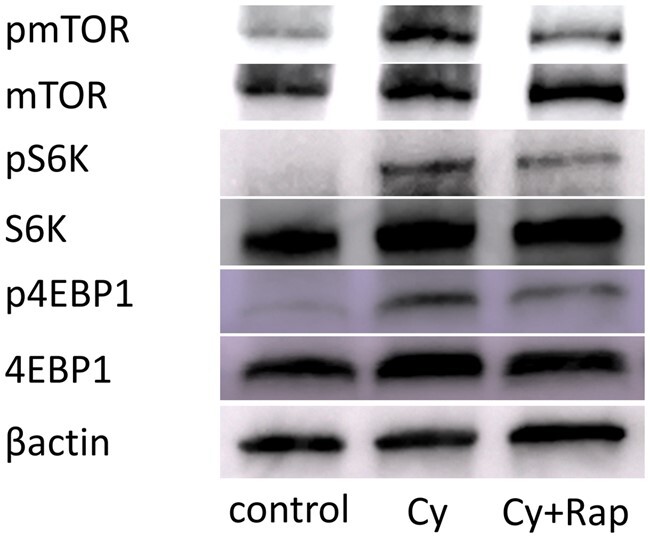
**Cyclophosphamide upregulates and rapamycin downregulates downstream targets of the mTOR pathway in the ovary**. Whole ovaries from mice treated with placebo or cyclophosphamide (Cy) or cyclophosphamide + rapamycin (Cy + Rap) were lysed for immunoblot. Representative results from western blotting are shown. In the Cy group, mTOR and downstream targets of the mTOR pathway (S6K and 4E-BP1) were more phosphorylated than control. In the Cy + Rap group, mTOR and downstream targets of the mTOR pathway were less phosphorylated than in the Cy group. S6K, S6 kinase.

**Figure 12.**
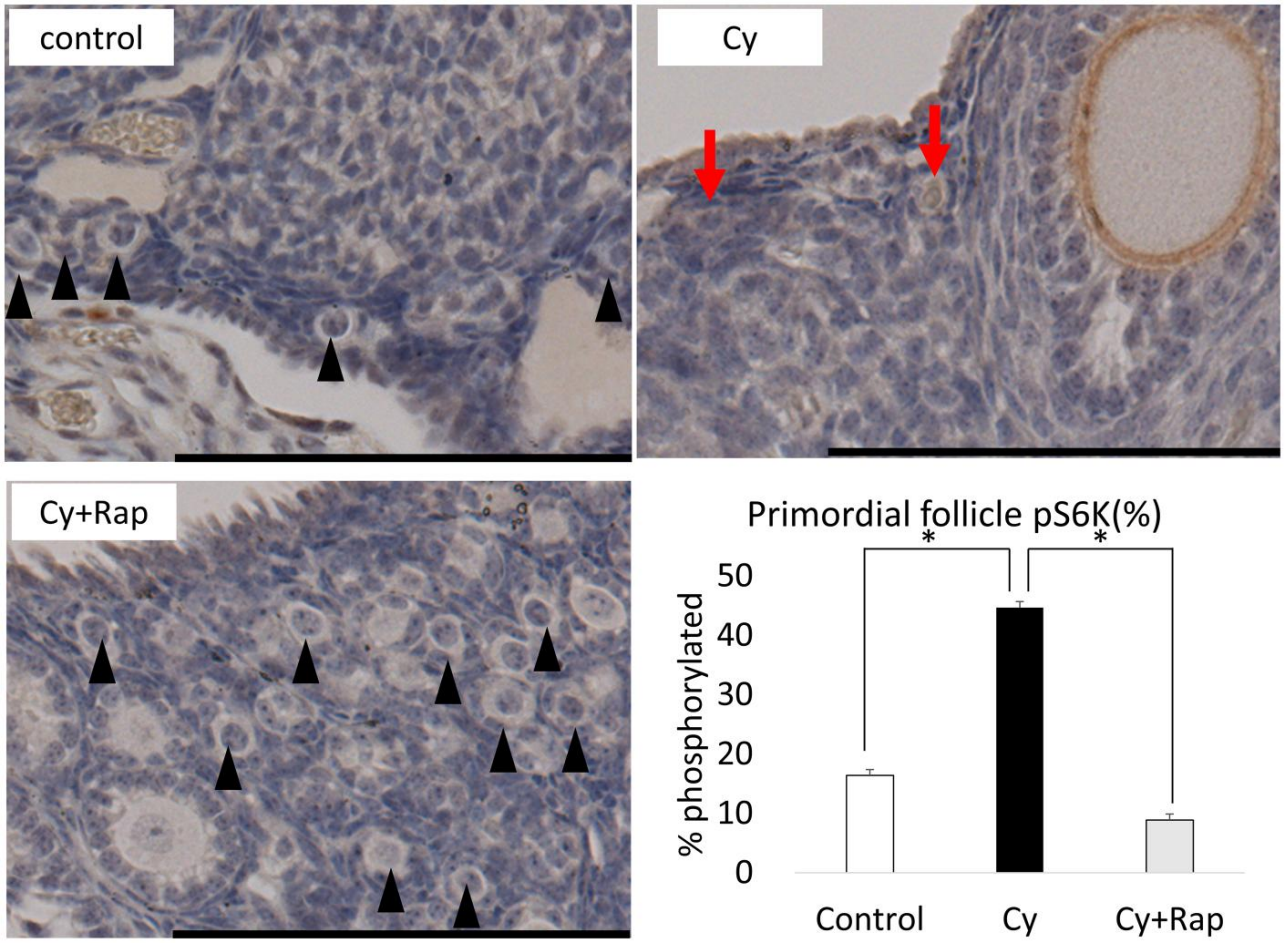
**Representative results from phosphorylated (p)S6K staining**. The control and Cy + Rap groups had many primordial follicles, most were pS6K negative. In contrast, there were fewer primordial follicles in the Cy group, but relatively more primordial follicles were pS6K positive. *P* < 0.001. n = 4–5/group. Bar = 100 μm. The data represent the mean ± SEM values. Cy, cyclophosphamide; Rap, rapamycin; S6K, S6 kinase.

Western blotting showed that mTOR, S6K, and 4E-BP1 (located downstream of the mTOR pathway) were activated by cyclophosphamide and inhibited by rapamycin (Fig. 11). Thus, rapamycin is supplied to the ovary and elicits an inhibitory effect against mTOR. Moreover, the pS6K-positive primordial follicle rate in the Cy group was 2.7 times higher than that in the control group. This effect was suppressed in the Cy + Rap group to a level that did not differ significantly from that of the control group (Fig. 12). Hence, cyclophosphamide locally activates the mTOR pathway, particularly in the primordial follicle rather than in the ovarian medulla. Meanwhile, rapamycin inhibits cyclophosphamide-induced primordial follicle activation via mTOR inhibition.

## Discussion

In this study, we realized an *in vivo* murine xenograft breast cancer model. Breast cancer is classified into four subtypes according to the positivity or negativity of hormone receptors (oestrogen and progesterone receptors) and HER2. In this study, triple-negative breast cancer (hormone receptor negative, HER2 negative) was selected as the subtype because, although triple-negative breast cancer is a minor subtype among all breast cancer cohorts, it is more common in younger breast cancer (Colleoni *et al.*, 2002; Futamura and Yoshida, 2022). Moreover, despite the fact that patients with triple-negative breast cancer have a high recurrence rate compared with non-triple-negative breast cancer (Costa *et al.*, 2018), specific endocrine therapies and targeted therapies are ineffective. Therefore, cytotoxic chemotherapy, primarily cyclophosphamide-based multi-combination chemotherapy, has become the main strategy for treating triple-negative breast cancer (Yin *et al.*, 2020). Additionally, in oestrogen receptor-positive breast cancer, if ovarian function is preserved by ovarian protection agents, blood oestradiol levels may increase and cause a tumour-enhancing effect on the transplanted breast cancer. However, in triple-negative breast cancer, ovarian protection with the preservation of ovarian function and elevated blood oestradiol levels are unlikely to cause tumour progression.

We also used immunodeficient mice to reproduce ovarian dysfunction in a carcinoma-bearing state. BALB/c nude mice were selected as they are only T-cell deficient and near-normal immunodeficient, compared with new strains of immunodeficient or severe combined immunodeficiency mice. Notably, cellular immunodeficiency (i.e. T-cell immunodeficiency) is associated with human breast cancer (Hadden, 2003). Given that the prognosis of breast cancer does not differ considerably between preoperative and postoperative chemotherapy (Early Breast Cancer Trialists’ Collaborative Group, 2018), the incidence of preoperative chemotherapy, particularly involving cyclophosphamide, is steadily increasing. Hence, our study, conducted in a xenograft mouse model, mirrors this evolving clinical scenario.

In the present study, doses and treatment cycles were selected based on those used in human clinical settings and mouse experimental models. In humans, a minimum of 9 months is required from the primordial follicle to ovulation (Zhou *et al.*, 2017). Cyclophosphamide, typically prescribed for neoadjuvant and adjuvant chemotherapy, is administered in a tri-weekly cyclic schedule, often spanning 4–6 cycles, all within a minimum of 9-month timeframe. In this regard, we considered a single dose inappropriate for replicating the human clinical model and opted for cyclic dosing. Cyclophosphamide was administered weekly, considering that the time between primordial follicle formation and ovulation was 3 weeks in mice (Zhou *et al.*, 2017).

**Figure 13.**
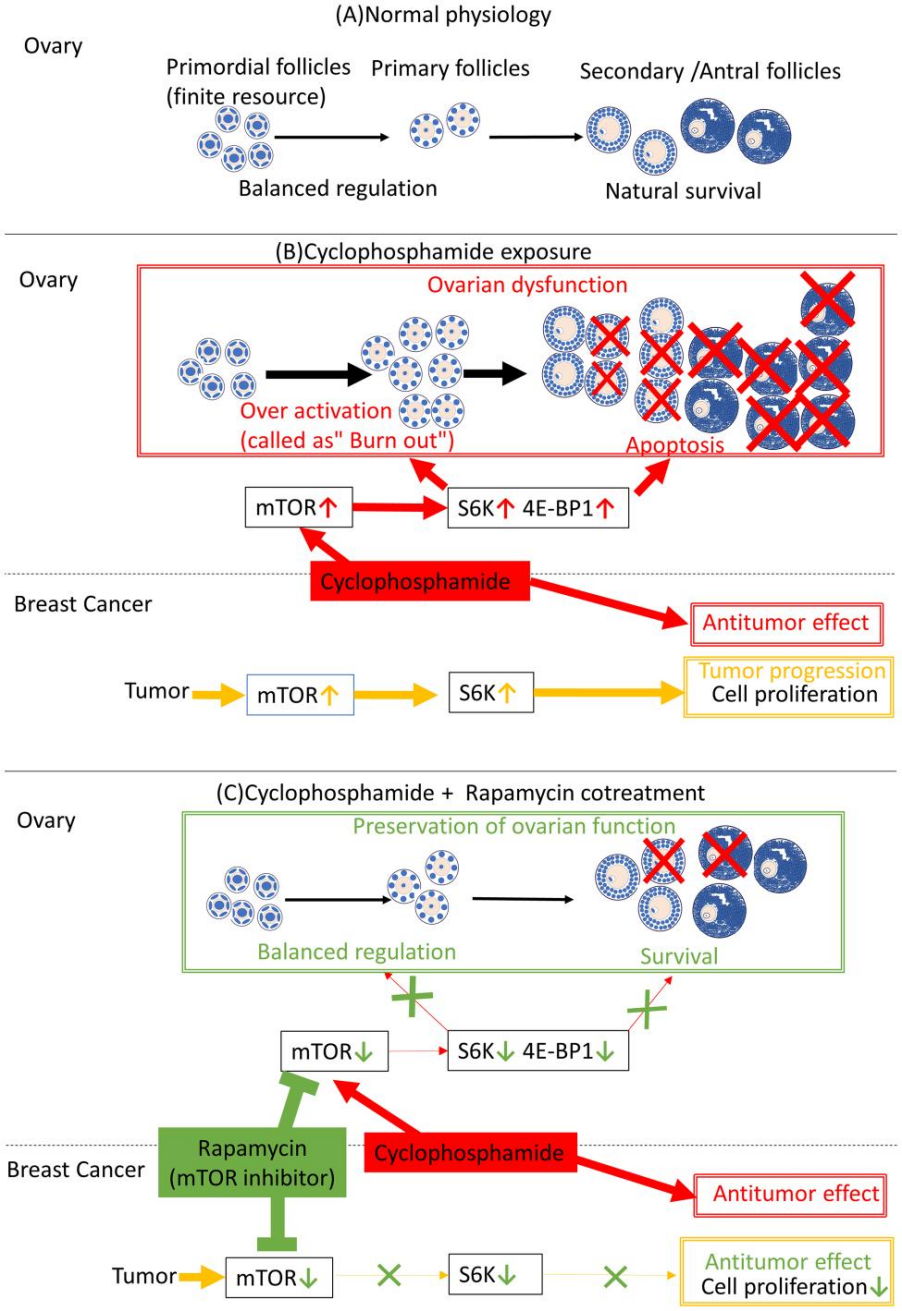
**Effects of cyclophosphamide and rapamycin on breast cancer and ovary**. (**A**) Normal physiology; the primordial follicle is a finite resource and is activated into a primary follicle by balanced regulation. The growing follicle has natural survival and does not undergo apoptosis. (**B**) When cyclophosphamide is administered to breast cancer patients, the primordial follicle is over-activated through the mTOR pathway, resulting in the apoptosis of many growing follicles and loss of the primary follicle. In breast cancer, activation of mTOR induces tumour progression through cell proliferation. Cyclophosphamide, as an alkylating anticancer drug, exerts a strong antitumour effect on breast cancer through the mechanism of DNA damage. (**C**) Cyclophosphamide + rapamycin cotreatment prevents loss of primordial follicles via rapamycin-induced mTOR pathway blocking. Rapamycin has the potential to inhibit cell proliferation by blocking mTOR activation in breast cancer.

Follicle activation occurs via phosphoinositide 3-kinase (PI3K)/AKT/mTOR pathway activation, resulting in the accumulation of numerous growing follicles, many of which undergo apoptosis through the PI3K/AKT/mTOR pathway (Kalich-Philosoph *et al.*, 2013; Qin *et al.*, 2022). The activation of AKT/mTOR by cyclophosphamide and the FOXO3a signalling network leads to FOXO3a export from the nucleus into the cytoplasm, promoting cellular proliferation (Liu *et al.*, 2007; Jang *et al.*, 2016). Moreover, γH2AX becomes elevated when the AKT/mTOR pathway is activated, whereas high intracellular levels of AKT/mTOR reportedly increase DNA damage, resulting in apoptosis (Bellusci *et al.*, 2019). The present study demonstrates that rapamycin exerts a protective effect against cyclophosphamide-induced primordial follicle loss. The mechanism of cyclophosphamide-induced follicle loss was found to be due to excessive primordial follicle development (so-called ‘burn out’), whereas rapamycin inhibits this effect, as evidenced by the ratio of primary to primordial follicles. Western blotting and immunostaining results further revealed that cyclophosphamide activated the mTOR pathway locally in primordial follicles, whereas rapamycin inhibited this effect. However, TUNEL staining 24 h after cyclophosphamide administration showed that primordial follicles do not directly undergo apoptosis, but rather granulosa cells of the growing follicles; this is inhibited by rapamycin (Fig. 13). Meanwhile, the smaller ovarian size in the Cy + Rap group may primarily reflect the fewer growing follicles, which comprise a larger proportion of the ovarian volume. Similarly, a previous study on the preventive effect of rapamycin on age-related follicle loss found more primordial follicles and fewer growing follicles, resulting in smaller ovaries in the rapamycin group compared with the control group (Dou *et al.*, 2017). In the Cy + Rap group, the number of growing follicles was low despite the prevention of apoptosis in growing follicles. This finding may be due to the rapamycin-mediated prevention of primordial follicle activation outweighing the rapamycin-mediated prevention of apoptosis in the growing follicle. Notably, activation of the mTOR pathway continued 7 days after cyclophosphamide administration. In mice, 3 weeks pass between the primordial follicle development and ovulation. In our study, the cyclophosphamide-induced primordial follicle activation persisted for more than one-third of the ovulation cycle, suggesting that mTOR inhibitors should be taken orally for an extended period after cyclophosphamide administration. Moreover, the residual toxicity from cyclophosphamide 1 week after the weekly regimen suggests an accumulation of cyclophosphamide ovarian toxicity.

Whether cyclophosphamide directly induces primordial follicle apoptosis is debateable. Although some authors claim the direct loss of primordial follicles following cyclophosphamide administration (Ganesan and Keating, 2016; Luan *et al.*, 2019; Nguyen *et al.*, 2019b), most investigators are of the opinion that over-activation of the primordial follicle (‘Burn out’) is the primary mechanism (Kalich-Philosoph *et al.*, 2013; Goldman *et al.*, 2017; Kano *et al.*, 2017; Lande *et al.*, 2017; Roness *et al.*, 2019; Sonigo *et al.*, 2019; Shai *et al.*, 2021). Meanwhile, Kashi *et al.* (2023) demonstrated that activation of the primordial follicle is largely responsible, with a small amount of direct DNA damage. The discrepancy in results likely stems from the time at which activation and apoptosis of the follicle occur. DNA damage and apoptosis by cyclophosphamide occur in 12–24 h, whereas activation of the primordial follicle is observed 24 h to several days later (Kalich-Philosoph *et al.*, 2013; Goldman *et al.*, 2017; Zhou *et al.*, 2017; Chen *et al.*, 2022a; Kashi *et al.*, 2023). There is also the question of which indexes (e.g. γH2AX, TUNEL, cPARP, or caspases) are most suitable for evaluating the direct damage and apoptosis caused by cyclophosphamide in primordial follicles. In the present study, we evaluated apoptosis by TUNEL staining 24 h after cyclophosphamide administration and found that apoptosis did not occur in primordial follicles. We also evaluated the mTOR pathway and primordial follicle activation 7 days after cyclophosphamide administration and observed that primordial follicle activation occurs via the mTOR pathway. Hence, the results of this study suggest that the loss of primordial follicles by cyclophosphamide is primarily due to activation of primordial follicles via the mTOR pathway.

mTOR inhibitors exert potential antitumour effects against certain subtypes of breast cancer. The level of evidence in human clinical practice is particularly high for hormone-receptor-positive breast cancer. A phase III randomized controlled trial study reported the efficacy and safety of mTOR inhibition with hormone-receptor-positive breast cancer (Piccart *et al.*, 2014). Meanwhile, probable antitumour effects of mTOR inhibitors have also been reported in triple-negative breast cancer. In triple-negative breast cancer, 72% of the tumours harbour phosphorylated mTOR, which is associated with shorter overall- and recurrence-free survival rates for patients with early-stage triple-negative breast cancer (Ueng *et al.*, 2012). Phosphorylation reactions stimulated by the PI3K/AKT/mTOR are responsible for cancer cell growth, proliferation, angiogenesis, tumour metastasis, and invasion (Arcaro and Guerreiro, 2007). Indeed, considerable *in vivo* and *in vitro* evidence have shown the antitumour efficacy of mTOR inhibitors as single agents or as sensitizing drugs with standard care drugs, including cyclophosphamide, and numerous studies, including our own, have reported that a main antitumour mechanism of mTOR inhibitors is their inhibitory effect on cell proliferation, as evidenced by employed Ki-67 immunostaining (Akcakanat *et al.*, 2009; Montero *et al.*, 2014; Zeng *et al.*, 2010; Zhang *et al.*, 2012, 2014; Eloy *et al.*, 2016; Wan *et al.*, 2016; Dankó *et al.*, 2021; Tan *et al.*, 2023). In this study, there was no statistically significant difference in tumour volume between the cyclophosphamide group and cyclophosphamide plus mTOR inhibitor group. The efficacy of anticancer drugs varies depending on the genetic profile of the cell line. MDA-MB-231, the cell line employed in this study, is sensitive to rapamycin *in vivo* (Zhang *et al.*, 2012; Wander *et al.*, 2013) but less sensitive than other triple-negative breast cancer cell lines (Liu *et al.*, 2011; Yunokawa *et al.*, 2012), suggesting that other triple-negative breast cancer cell lines may exhibit a stronger tumour volume suppression effect following rapamycin treatment. Hence, rapamycin, at the minimum, does not weaken the anticancer effect of cyclophosphamide against triple-negative breast cancer and may augment an anti-tumour cell proliferation effect (Fig. 13). However, the lack of statistically significant differences in tumour volume prevented the confirmation that rapamycin elicits an antitumour effect on breast cancer. Prior to the clinical application of mTOR inhibitors for antitumour effects, phase III trials assessing their potential against triple-negative breast cancer are warranted.

Mucositis and stomatitis are the most commonly reported adverse effects of mTOR inhibitors (Nguyen *et al.*, 2019a). In this study, no obvious stomatitis was observed during autopsy, and no weight loss due to mTOR inhibitors was observed, suggesting that feeding difficulties due to stomatitis were negligible.

A limitation of this study is that although rapamycin elicits follicle-protective effects, it cannot fully prevent the follicle loss induced by cyclophosphamide. Nevertheless, the advantages of rapamycin as a follicle protective agent can be exploited in combination with hormone therapy, recombinant anti-Müllerian hormone (Kashi *et al.*, 2023), or a GnRH agonist. Combination therapy with stem cells is also expected to be effective (Takahashi *et al.*, 2021). In combination with other mechanism-based agents, mTOR inhibitors may exhibit more complete follicle protective against high doses of cyclophosphamide.

Additionally, we were unable to examine follicle functionality and fertility in this study. Although two studies have evaluated litter size after cyclophosphamide and mTOR inhibitor co-treatment as ovarian protectants (Goldman *et al.*, 2017; Kashi *et al.*, 2023), both studies evaluated litter size 8–16 weeks after termination of anticancer therapy. If the present experimental model is prolonged and matching is performed for 8 weeks after cyclophosphamide and rapamycin completion, a humane endpoint will likely be reached since the mice will be followed for several months without administration of antitumour drugs in the cancer-bearing state. Thus, new protocols are needed to evaluate follicle functionality and fertility and antitumour effects in a single experimental model. For example, using the breast cancer xenograft mouse model, antitumour effects can be measured after administering rapamycin and cyclophosphamide to mimic neoadjuvant chemotherapy. Tumour resection should be performed at the end of Cy and rapamycin treatment, and with the litter size evaluated after a matching protocol.

In conclusion, this study demonstrated that mTOR inhibitors exert an ovario-protective effect during cyclical cyclophosphamide regimens, which have high antitumour effects as single agents in breast cancer. In terms of antitumour efficacy, although there was no statistically significant difference in tumour volume, our results suggest that mTOR inhibitors could potentially inhibit tumour proliferation.

## Data Availability

The data in this article are available in the article, and when necessary by request from yujit@belle.shiga-med.ac.jp.
